# Growth‐associated protein 43 promotes thyroid cancer cell lines progression via epithelial‐mesenchymal transition

**DOI:** 10.1111/jcmm.14460

**Published:** 2019-09-30

**Authors:** Chen Zheng, Rui‐Da Quan, Cheng‐Yong Wu, Jing Hu, Bang‐Yi Lin, Xu‐Bing Dong, Er‐Jie Xia, Adheesh Bhandari, Xiao‐Hua Zhang, Ou‐Chen Wang

**Affiliations:** ^1^ Department of Thyroid & Breast Surgery The First Affiliated Hospital of Wenzhou Medical University Wenzhou People’s Republic of China

**Keywords:** EMT, GAP43, lymph node metastasis, oncogene, papillary thyroid cancer

## Abstract

Thyroid cancer is maintaining at a high incidence level and its carcinogenesis is mainly affected by a complex gene interaction. By analysis of the next‐generation resequencing of paired papillary thyroid cancer (PTC) and adjacent thyroid tissues, we found that Growth Associated Protein 43 (GAP43), a phosphoprotein activated by protein kinase C, might be novel markers associated with PTC. However, its function in thyroid carcinoma has been poorly understood. We discovered that GAP43 was significantly overexpressed in thyroid carcinoma and these results were consistent with that in The Cancer Genome Atlas (TCGA) cohort. In addition, some clinicopathological features of GAP43 in TCGA database showed that up‐regulated GAP43 is significantly connected to lymph node metastasis (*P* < 0.001) and tumour size (*P* = 0.038). In vitro experiments, loss of function experiments was performed to investigate GAP43 in PTC cell lines (TPC‐1 and BCPAP). The results proved that GAP43 knockdown in PTC cell significantly decreased the function of cell proliferation, colony formation, migration, and invasion and induced cell apoptosis. Furthermore, we also indicated that GAP43 could modulate the expression of epithelial‐mesenchymal transition‐related proteins, which could influence invasion and migration. Put those results together, GAP43 is a gene which was associated with PTC and might be a potential therapeutic target.

## 1 INTRODUCTION

Thyroid cancer (TC) is maintaining at a high incidence level and it becomes one of the most common cancers in worldwide.^1^, ^2^ The US National Cancer Institute anticipated 53 990 new cases besides 2110 death numbers of patients because of TC in the USA in 2018.^3^, ^4^ Besides, the papillary thyroid cancer (PTC) is the commonest sub‐type of TC (accounts for 80%‐90%).^5^ Although the incidence of PTC is high, it is generally considered that PTC is relatively curable and has a good prognosis.^6^, ^7^ After a surgical operation or radio‐iodinated therapy, patients with PTC show satisfactory prognosis besides a general 10‐year survival rate of 90%.^7^ However, clinical course of PTC usually follows indolent and carries excellent prognosis, it is still highly metastatic and relapses after routine treatment.^5^ Lymph node metastasis (LNM) is the main factor of locoregional recurrence and a distinct risk factor of mortality.^8^, ^9^, ^10^ LNM has been shown to occur in approximately 20% of all PTC patients, and regional recurrence occurred in 10% of patients undergoing total thyroidectomy.^11^ So, it is vital for us to facilitate the in‐depth investigation of the mechanism of PTC and help doctors to provide an appropriate treatment approach.

The incidence and development of PTC are primarily affected by genomic alternation, involving stimulation of oncogene and silencing of a tumour suppressor gene. Accumulating reports have been published about molecular mechanisms of PTC over nearly a period of 20 years. B‐type Raf kinase (BRAF) V600E, a famous gene mutation, promotes PTC tumorigenesis and progression by abnormally stimulating the mitogen‐activated pathway kinase pathway.^12^ Besides, some notable mutations such as RAS mutation,^13^ TERT mutation,^14^ PTEN mutation,^15^ PIK3CA mutation^16^, ^17^ and TP53 mutation^18^ also show a significant part in thyroid carcinoma. Numerous researchers have made remarkable progress in TC gene research, but many characters of PTC are yet relatively unspecified. Hence, examining for new potential markers and clarifying molecular mechanisms in the improvement of thyroid carcinoma is still essential.

In our previous study, we have conducted next‐generation of 19 pairs of PTC tissues and adjacent normal thyroid tissue.^19^ After evaluating all the information, we identified that growth‐associated protein 43 (GAP43), a gene encoding an axonal membrane protein, as an oncogene promotes PTC tumorigenesis and tumour progression.^19^ As for the normal function of this protein, it is normally expressed in neuronal somata, axons and growth cones during pre‐ and early postnatal development.^20^, ^21^ Consistently, it also plays a fundamental function in neural growth, axonal regeneration and stabilisation of synaptic function.^22^, ^23^ A study indicated that GAP43 genetic variants and its gene network interaction may associate with the susceptibility to Hirschsprung disease.^24^ A considerable report of the mechanisms indicated that GAP43 may promote non‐small lung cancer (NSLC) cell migration by activating Rac1 and mediating F‐actin cytoskeleton polymerisation.^25^ Emerging shreds of evidence have manifested that GAP43 has an important function in many aspects; however, whether the GAP43 gene also plays a critical role in PTC remains unclear.

To evaluate the mRNA expression profiles of PTC, 19 paired PTC samples and adjacent non‐carcinomatous samples were conducted to whole transcriptome sequencing. We firstly discovered GAP43 was significantly overexpressed in PTC tissue. Hence, we collected 50 pairs of tumour samples and adjacent normal tissues to validate the expression of GAP43 by using quantitative reverse transcription‐polymerase chain reaction (qRT‐PCR). Moreover, we also analysed the relationship of GAP43 expression and clinical features in PTC. Then, we performed a series of cell line experiments and Western blot by siRNA to explore the role of GAP43 in TC. We established that loss of function about GAP43 effectively inhibits the proliferation and invasion of two PTC cell lines (TPC‐1, BCPAP) and promotes apoptosis in vitro. Down‐regulation of GAP43 in those cell lines decreased the N‐cadherin and vimentin and increased E‐cadherin.

All in all, we firstly proved that GAP43 has a potential role in PTC cell progression and could be a novel target in PTC patients.

## 2 MATERIALS AND METHODS

### 2.1 Samples collection

As a validated cohort, we gathered 50 fresh PTC and paired normal tissues from patients through surgery at the First Affiliated Hospital of Wenzhou Medical University between 2014 and 2018. All those patients did not acquire chemotherapy or radiotherapy for pre‐treatment. The samples were instantly achieved at the time of initial surgery and were frozen in liquid nitrogen instantly after lesion resection and then stored at −80 Celsius before RNA extraction. All tumour tissues were histologically reviewed by two pathologists, and the cases were retrospectively reviewed by two senior pathologists to confirm the histological diagnosis. Informed consent for the scientific use of the biological material was obtained from each patient. All patient‐derived information was recorded following the protocols approved by the ethical standards of the Ethics Committee of the First Affiliated Hospital of Wenzhou Medical University (approval no. 2012‐57).

### 2.2 The Cancer Genome Atlas (TCGA) database

A total of 502 PTC patients with complete clinicopathological characteristics (such as age, sex, LNM, tumor size, clinical stage (ACJJ7), and histological type) were collected for further analysis. All data were downloaded from the TCGA data portal (https://tcga-data.nci.nih.gov/docs/publications/tcga/).

### 2.3 RNA isolation and real‐time reverse transcription/polymerase chain reaction (qPCR)

The total RNA from the tissues was isolated using TRIzol reagent (Invitrogen; Thermo Fisher Scientific, Inc), according to the manufacturer's protocol (Life Technologies, Carlsbad, CA), cDNA was prepared using a ReverTra Ace qPCR RT kit (Toyobo, Osaka, Japan). To evaluate the RNA quality and quantity, we used the A260/A280 ratio as the control value. cDNA samples were all stored at −80°C. Then, the relative expression of GAP43 was detected by ABI Prism 7500 Sequence Detection System (Thermo Fisher Scientific) by One Step SYBR® PrimeScript®PLUS

RT‐RNA PCR Kit (TaKaRa, Tokyo, Japan) according to the user guide protocol. The primer sequences for PCR are as follows: GAP43, forward 5′‐ TTCCGTGGACACATAACAA‐3′ and reverse 5′‐ GGACATCATCCTTCTTCTCT‐3′; GAPDH, forward 5′‐GGTCGGAGTCAACGGATTTG‐3′ and reverse 5′‐ATGAGCCCCAGCCTTCTCCAT‐3′.

### 2.4 Cell culture and growth conditions

The three types of human PTC cell line (TPC‐1, KTC‐1 and BCPAP) and normal thyroid (HTORI3) cell lines were obtained from the Chinese Academy of Sciences Stem Cell Bank. The use of the above cell lines has been approved by the Ethics Committee of The First Affiliated Hospital of Wenzhou Medical University. These cell lines all were cultured in RPMI 1640 (Invitrogen; Thermo Fisher Scientific, Inc, Waltham, MA, USA), supplemented with 10% foetal bovine serum (FBS; Invitrogen; Thermo Fisher Scientific, Inc), 1 × MEM non‐essential amino acids and 1 × sodium pyruvate. All cells were maintained in a humidified incubator at 37°C with 5% CO_2_. TPC‐1, KTC1 and BCPAP cells were plated onto six‐well plates at concentrations of 4.5 × 10^5^, 7.5 × 10^5^ and 8 × 10^5^ cells/ well respectively, and incubated for 24 hours in the growth medium.

### 2.5 RNA interference

For cell interference, siRNA for GAP43 was synthesised from Shanghai Gene Pharma (Shanghai, China). The sequences of the GAP43 and negative control siRNA are as follows: (Sense‐1: GCCGUCCUCCAAGGCUGAATT; Antisense‐1: UUCAGCCUUGGAGGACGGCTT; Sense‐2: GCCAAGCUGAAGAGAACAUTT; Antisense‐2: AUGUUCUCUUCAGCUUGGCTT). Scramble sequences were used as negative controls (Si‐NC) in our experiments. The scramble sequences are randomly added without any target sequence tracks. 5 × 10^4^ TPC or 6 × 10^5^ BCPAP cells were transfected with either 10 µL siRNA (TPC‐1) or 5 µL siRNA (BCPAP) of iMAX (Life Technologies, Carlsbad, CA, USA) according to the company's instructions. Cells were harvested 48 hours after transfection for further experiments or analysis. All knockdown experiments were repeatedly performed three times.

### 2.6 Cell proliferation assay

A Cell Counting Kit‐8 (CCK‐8, Beyotime, China) was assessed to observe cell proliferation ability according to the company's protocol. Cells were seeded into 96‐well plates at the density of 1.5 × 10^3^ cells per well after transfection. Subsequently, 10 μL CCK‐8 solution was added to each individual well. This same procedure was repeatedly measured at 1, 2, 3 and 4 days, respectively. CCK‐8 solution (10 μL) was added to each well and incubated for 2.5 hours at 37°C. Absorbance levels were detected at 450 nm (OD450) using SpectraMax M5 (Molecular Devices LLC, Sunnyvale, CA, USA). For every group, data from five different wells were pooled. All the assays were performed in triplicate.

### 2.7 Colony formation assay

The two transfected or control groups were planted into six‐well plates at 1.5 × 10^3^ cells and preserved in RPMI 1640 containing 10% FBS for 8‐14 days. Afterwards, the cells were rinsed with phosphate‐buffered saline (PBS), fixed with 4% PFA (paraformaldehyde Sigma, USA) for 30 minutes. Finally, plates were stained with crystal violet (Sinopharm Chemical Reagent, Beijing, China). All the assays were performed in triplicate.

### 2.8 Migration and invasion assays

In the migration assays, we used Transwell chambers (Corning Costar Corp, Cambridge, MA, USA). Two PTC Cells (3 × 10^4^ for TPC‐1, 3.5 × 10^4^ BCPAP) were suspended in 300 µL non‐serum RPMI1640 and plated into the upper chamber. For the lower chamber, 600 µL of RPMI1640 including 10% FBS was filled in a 24‐well plate. Next, the cells were incubated in the above condition. After 24 hours, the cells adhered to the upper chamber were erased by cotton swabs, and the migration cells on the lower chamber were secured and stained with 0.4% crystal violet. We used Matrigel Invasion Chambers (Corning, NY, USA) as invasion assays. The same technique described for the migration assay was done with the invasion chambers.

### 2.9 Flow cytometry

An Annexin V/propidium iodide (PI) apoptosis kit (Nanjing KeyGen Biotech. Co., Ltd., Nanjing, China) were used to determine the apoptotic rates of TPC1, BCPAP according to the company's advice. The cells were collected, rinsed three times by PBS, and suspended in 1 × binding buffer (Beyotime Institute of Biotechnology, Haimen, China) at a concentration of 1 × 10^6^ cells/mL. Cell suspensions of 300 µL were stained with 5 µL of Annexin V‐fluorescein isothiocyanate and 5 µL of PI at room temperature for 15 minutes in the darkroom prior to analysis by flow cytometry (BD FACS Aria; BD Biosciences, Franklin Lakes, NJ).

### 2.10 Protein extraction and Western blot analysis

The protein of transfected cells lysates was lysed in cell lysis buffer (Beyotime, China). Protein concentrations were measured using a bicinchoninic acid assay (BCA). By using SDS‐PAGE on a 10% gel and electrotransferred to PVDF membranes total proteins (20 μg) in the lysate were removed. Using 5 % skimmed milk (BD, Difco™ Skim Milk, 232100), the membranes were blocked for 2 hours and incubated by primary antibodies overnight at 4°C. After rinsing three times with triple buffered saline and Tween 20, the membranes were incubated for 2 hours with secondary antibody at normal room temperature. After rinsing three times with triple buffered saline and Tween 20, the cells were incubated for 1.5 hours with secondary antibody at room temperature. All band intensities were quantified by Image Lab software. The primary antibodies used included vimentin (Abcam), N‐cadherin (Abcam), E‐cadherin (Abcam), GAP43 (Abcam) and β‐actin (Sigma). Goat anti‐rabbit HRP‐conjugated IgG (Abcam) and goat anti‐mouse HRP‐conjugated IgG (Abcam) as the secondary antibody β‐actin served as an internal control.

### 2.11 Statistical analysis

Statistical data evaluations were accomplished using SPSS 23.0 software (SPSS, Inc, Chicago, IL). GraphPad Prism7 (GraphPad Software, Inc, La Jolla, CA, USA) was used for creating the graphs. Data on normal distribution were expressed as mean ± standard deviation. Otherwise, the differences between treated and control groups were estimated by Student’s *t*‐test (two‐tailed). As for the clinical feature, patients of PTC were divided into two parts based on the high and low expression level of GAP43, with the median value as the cutting line. *P* < 0.05 was considered to indicate a statistically significant difference.

## 3 RESULTS

### 3.1 GAP43 overexpression in PTC

**Figure 1:**
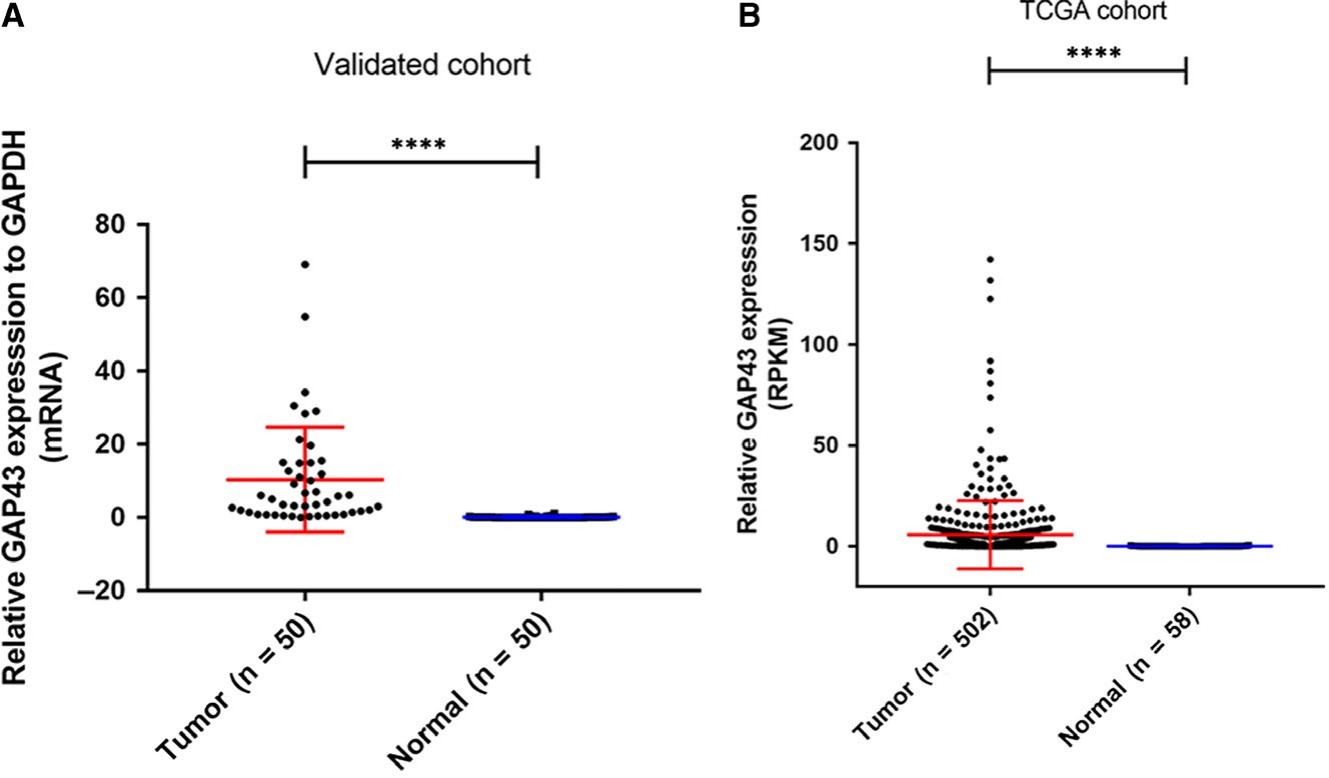
The mRNA expression of GAP43 both in local validated cohort and TCGA cohort. A, GAP43 expression was significantly up‐regulated in PTC tissues compared with the adjacent non‐cancerous thyroid tissues using qRT‐PCR (****P < 0.0001). B, Data including 502 PTC samples and 58 adjacent non‐cancerous thyroid samples, GAP43 expression was also significantly down‐regulated in PTC in TCGA cohort (****P < 0.0001). Abbreviations: GAP43, growth‐associated protein 43; PTC, papillary thyroid carcinoma; qRT‐PCR, quantitative reverse transcription‐polymerase chain reaction; TCGA, The Cancer Genome Atlas

To investigate the function of GAP43 in PTC, we conducted 19 pairs of primary PTC tissues and adjacent normal tissues via whole transcriptome sequencing. After analysing this data, we identified GAP43 as up‐regulated in most PTCs (Table 1). Furthermore, we detected the mRNA expression level of GAP43 in 50 patients via RT‐qPCR to certify our sequencing results. In tumour tissues, expression level of GAP43 was significantly higher comparing with adjacent non‐tumour tissue (Figure 1A). This result is consistent with the data revealed in the TCGA cohort (Figure 1B).

**Table 1:** The expression of growth associated protein‐43 gene in 19 cases of thyroid papillary carcinoma was higher than that in normal tissue by whole transcriptome sequencing

Symbol	RN‐FPKM	RT‐FPKM	Log 2 ratio (RT/RN)	RT/RN
GAP43	0.033936635	1.949363226	1.759223992	Up
GAP43	0	1.895330456		Up
GAP43	0.08772778	3.699854637	1.625047522	Up
GAP43	0	0.948829382		Up
GAP43	0.573899397	0.875060606	0.183202365	Up
GAP43	0.044775052	27.73978573	2.792067003	Up
GAP43	0.056147344	1.423116966	1.403911379	Up
GAP43	0.183203548	4.06582776	1.346215097	Up
GAP43	0.067258766	0.877797662	1.115645525	Up
GAP43	0.010895226	0.865856499	1.900209678	Up
GAP43	0.056025915	1.045908521	1.271104743	Up
GAP43	0.158057854	0.331310457	0.321419062	Up
GAP43	0.06891197	27.14887438	2.595457163	Up
GAP43	0.055769441	0.55906537	1.0010663	Up
GAP43	0.078664129	1.028488235	1.11642259	Up
GAP43	0.04452478	4.937265171	2.044884671	Up
GAP43	0.033434048	5.761393281	2.23633856	Up
GAP43	0.285280218	9.576019137	1.525913348	Up
GAP43	0.156508085	18.80984738	2.079848494	Up

Abbreviations: FPKM, fragments per kilobase million; GAP43, Growth Associated Protein‐43; RN, RNA normal tissues; RT, RNA tumour tissues.

### 3.2 GAP43 was associated with the clinicopathological features of PTC

To facilitate in‐depth investigation of GAP43 in PTC, the relationship of GAP43 and the clinicopathological features was further analysed. In TCGA cohort, we divided 502 patients into high GAP43 expression (n = 251) and low GAP43 expression (n = 251) groups according to the median value. Results showed that histological type (*P* < 0.001), tumour size (*P* = 0.038) and LNM (*P* < 0.001) were significantly related to high GAP43 expression (Table 2). Conversely, no significant associations were found between GAP43 expression and gender, age, distant metastasis, disease stage (AJCC7) and multi‐focality. Similarly, in our validated cohort, high GAP43 expression was correlated with the tumour size (*P* = 0.021) and LNM (*P* = 0.041), but disease stage (AJCC7) was insignificant (*P* = 0.774), as shown in Table 3. Overall, those results have shown that GAP43 might serve as a oncogene in PTC.

**Table 2:** The association between growth associated protein‐43 expression and clinicopathologic features in The Cancer Genome Atlas cohort

Clinicopathologic features	High expression (N = 251)	Low expression (N = 251)	X^2^	*P* value
Gender				
Female	184	183	0.01	0.92
Male	67	68		
Age (years)				
Mean ± SD	46.06 ± 15.16	48.62 ± 16.407	0.805	0.37
≤45	125	111		
>45	126	140		
Histological type				
Classical	204	152	26.116	<0.001
Other types	47	99		
Multi‐nodularity				
Yes	124	102	3.031	0.082
No	125	141		
Tumour size (mm)				
≥20	189	168	4.319	0.038
<20	61	82		
Lymph‐node metastasis				
Yes	137	86	21.276	<0.001
No	91	138		
Distant metastasis				
Yes	8	7	0.069	0.793
No	243	244		
Disease stage (AJCC7)				
I + II	159	174	2.023	0.155
III + IV	91	76		

Notes

Chi‐square test, *P*‐value <0.05.

Abbreviations: AJCC7, Seven Edition of American Joint Committee on Cancer; TCGA, The Cancer Genome Atlas.

**Table 3:** The Association between growth associated protein‐43 expression and clinicopathologic features in a validated cohort

Clinicopathologic features	High expression (N = 25)	Low expression (N = 25)	X^2^	*P* value
Gender				
Female	12	11	0.081	0.777
Male	13	14		
Age (years)				
Mean ± SD	44.56 ± 14.22	47.62 ± 15.54	0.082	0.774
<45	11	10		
≥45	14	15		
Multi‐nodularity				
Yes	12	11	0.081	0.777
No	13	14		
Tumour size (mm)				
≥20	19	11	5.333	0.021
<20	6	14		
Lymph‐node metastasis				
Yes	13	6	4.160	0.041
No	12	19		
Capsule invasion				
Yes	2	1		1
No	23	24		
Disease stage (AJCC7)				
I + II	15	14	0.082	0.774
III + IV	10	11		

Notes

Chi‐square test, *P*‐value <0.05.

Abbreviations: AJCC, American Joint Committee on Cancer; TCGA, The Cancer Genome Atlas.

### 3.3 High GAP43 expression intensifies the risk of LNM in PTC patients

Through logistic regression analysis, we assessed the relationship between GAP43 expression and LNM. We revealed that the significant variables for LNM were GAP43 expression (odds ratio [OR] = 2.462, 95% confidence interval [CI] = 1.678‐3.594, *P* < 0.001), age (OR = 0.62, 95% CI = 0.427‐0.889, *P* = 0.012), histological type (OR = 0.646, 95% CI = 1.544‐3.680, *P* < 0.001), tumour size (OR = 2.496, 95% CI = 1.619‐3.764, *P* < 0.001), gender (OR = 1.551, 95% CI = 1.022‐2.353, *P* = 0.039) and disease stage (OR = 3.493, 95% CI = 2.316‐5.268, *P* < 0.001; Table 4) in univariate logistic regression analysis. Meanwhile, multivariate logistic analysis validated that GAP43 expression (OR = 2.135, 95% CI = 1.371‐3.325, *P* = 0.001), age (OR = 0.29, 95% CI = 0.009‐0.097, *P* < 0.001), histological subtype (OR = 2.615, 95% CI = 1.575‐4.342, *P* < 0.001) and disease stage (OR = 60.855, 95% CI = 17.791‐208.165, *P* < 0.001) were significant and independent high‐risk factors of LNM by using all parameters (Table 5).

**Table 4:** Univariate logistic regression analysis for the risk of lymph node metastasis

Factor	OR	95% CI	*P*–value
GAP43 expression (high vs low)	2.462	1.678‐3.594	<0.001^a^
Histological type	2.383	1.544‐3.680	<0.001^a^
Age, years (≤45 vs >45)	0.62	0.427‐0.899	0.012^a^
Gender (male vs female)	1.551	1.022‐2.353	0.039^a^
Tumour size (mm)	2.469	1.619‐3.764	<0.001^a^
Disease stage (AJCC7)	3.493	2.316‐5.268	<0.001^a^
Multi‐nodularity	1.446	0.994‐2.103	0.054

Abbreviations: AJCC, American Joint Committee on Cancer; GAP43, Growth Associated Protein‐43.

a *P* value <0.05.

**Table 5:** Multivariate logistic regression analysis for risk of lymph node metastasis

Factor	OR	95% CI	*P*–value
GAP43 expression (high vs low)	2.135	1.371‐3.325	0.001^a^
Histological Type	2.615	1.575‐4.342	<0.001^a^
Age, years (≤45 vs >45)	0.29	0.009‐0.097	<0.001^a^
Gender (male vs female)	1.489	0.901‐2.462	0.12^a^
Disease stage (AJCC7)	60.855	17.791‐208.165	<0.001^a^
Tumour size (mm)	1.304	0.769‐2.211	0.325^a^

Abbreviations: AJCC, American Joint Committee on Cancer; GAP43, Growth Associated Protein‐43.

a *P* value <0.05.

### 3.4 GAP43 knockdown restrains TPC‐1 and BCPAP cell proliferation and colony formation

**Figure 2:**
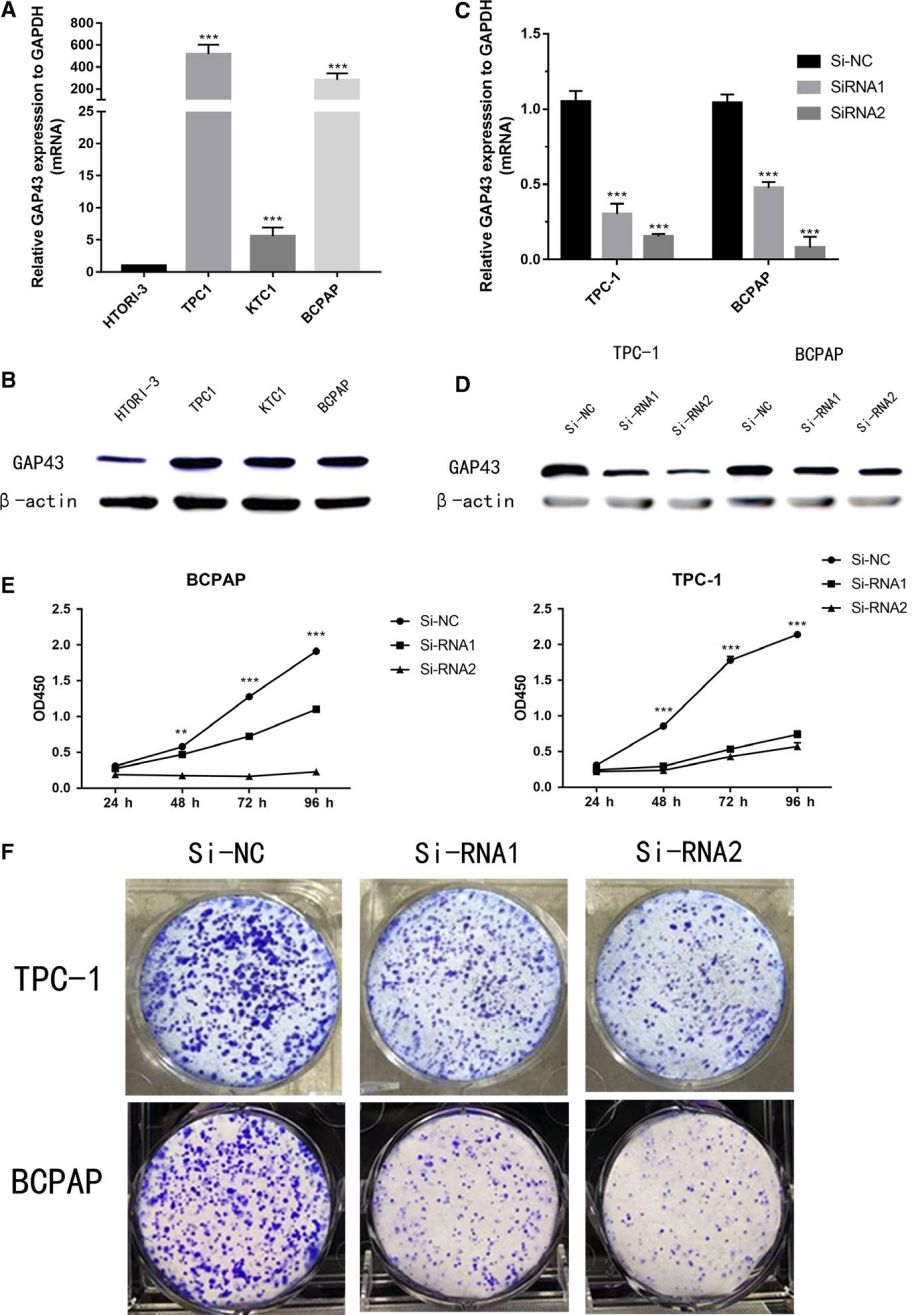
Down‐regulated expression of GAP43 promotes cell proliferation in PTC cell lines. A, B, The relative expression of GAP43 (compared with the GAPDH gene) was examined via RT‐qPCR and WB. GAP43 gene is expressed at higher levels in TPC‐1, KTC‐1 and BCPAP than HTORI3 (normal thyroid cell line). C, D, The relative expression of GAP43 (compared with the GAPDH gene) was verified by RT‐qPCR and WB in TPC‐1, BCPAP cell lines. Compared with the corresponding Si‐NC group, the expression of GAP43 in Si‐RNA group was lower. E, CCK‐8 assays: TPC‐1, BCPAP cell lines transfected with Si‐RNA1, Si‐RNA2 or Si‐NC were cultured in 96‐well plates for 1‐4 d and using CCK‐8 measured cell proliferation. F, Colony formation of Si‐RNA1, Si‐RNA2, and Si‐NC cells: Si‐RNA1, Si‐RNA2 significantly restrains cell proliferation in thyroid cell. **P < 0.01; ***P < 0.001 in comparison with the control group using Student's *t*‐test. Abbreviations: GAP43, growth‐associated protein 43; PTC, papillary thyroid carcinoma; RT‐qPCR, reverse transcription‐quantitative polymerase chain reaction

We also assessed the GAP43 expression level both in several PTC cell lines and normal thyroid cell lines (HTORI‐3) by using RT‐qPCR and WB. The results showed that GAP43 gene was commonly high‐expressed in TPC‐1, KTC‐1 and BCPAP (Figure 2A, 2B). Given the GAP43 gene is commonly overexpressed in PTC, we hypothesised GAP43 plays an oncogenic function in TC cell progression. Next, we selected TPC‐1 and BCPAP as relatively high‐expression cell lines to explore the function of GAP43. We down‐regulated the GAP43 expression level via effective small interfering RNA (siRNA‐1, siRNA‐2). Obviously, Si‐RNA could knock off the expression of GAP43 more than half (Figure 2C, 2D). Next, down‐regulation of GAP43 significantly inhibited TPC‐1 and BCPAP cell line proliferation as assessed by CCK‐8 and colony formation assays (Figure 2E, 2F).

### 3.5 Knockdown of GAP43 represses TPC‐1 and BCPAP cell migration and invasion in vitro

**Figure 3:**
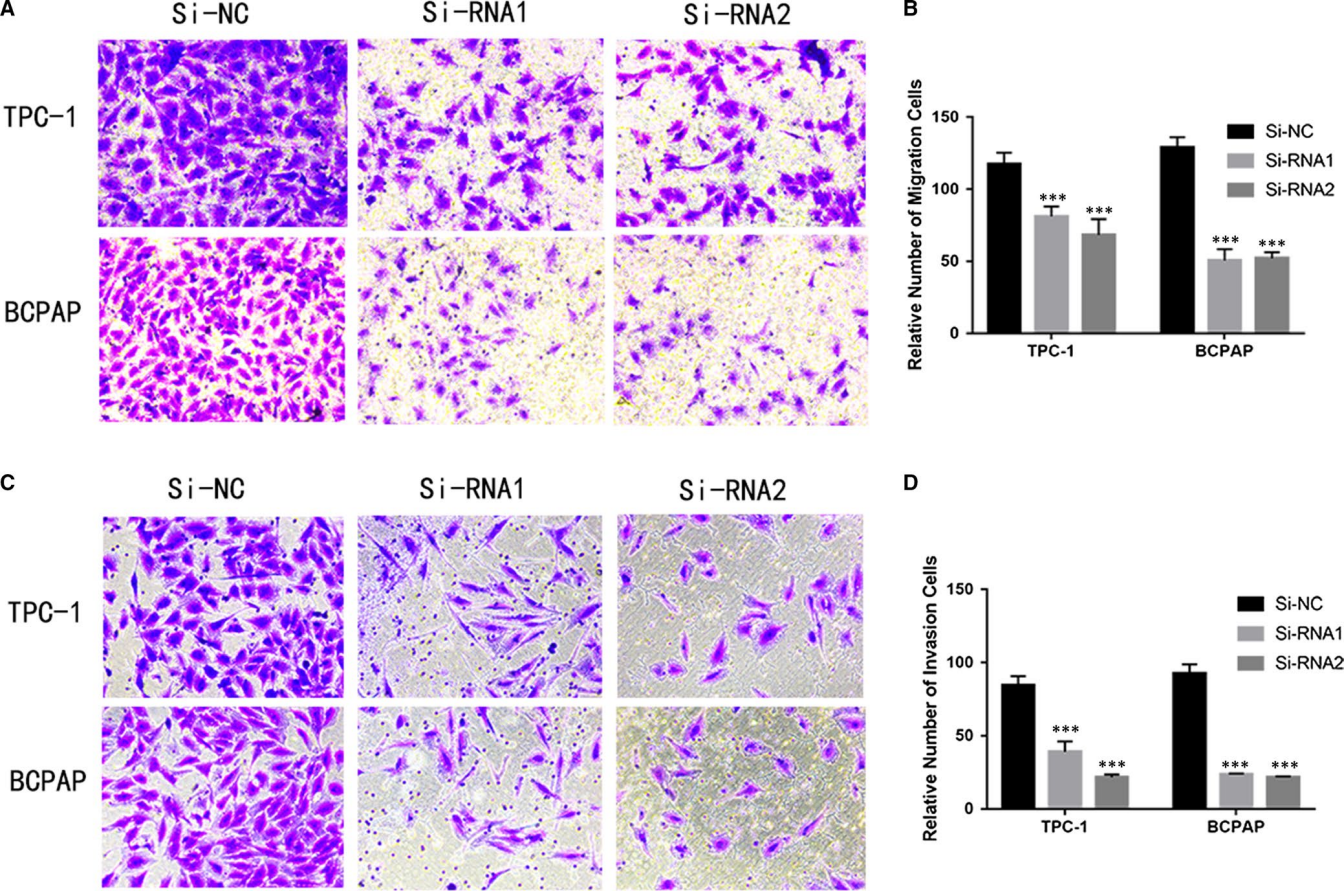
Knockdown of growth‐associated protein 43 (GAP43) represses TPC‐1 and BCPAP cell migration and invasion in vitro. A, C, Transwell migration and invasion assays in cells with down‐regulated GAP43 expression and their corresponding control cells. B, D, Quantitative results of migration and invasion assays. The stained cells were manually counted from five randomly selected fields and normalised with the observed cell proliferation. ***P < 0.001 in comparison with the NC group using Student's *t*‐test

Given the results of clinicopathological features analysis and cell proliferation and colony formation experiments, we further hypothesised the GAP43 might have a function on the PTC cell line migration and invasion. As predicted, the results showed that down‐regulated GAP43 significantly inhibited TPC‐1 and BCPAP migration capacity compared with the control groups (Figure 3A–3B). Similarly, knockdown GAP43 can significantly inhibit the cell invasive ability comparing with corresponding Si‐NC cell lines (Figure 3C–3D).

### 3.6 GAP43 gene knockdown induces the cell apoptosis of PTC cell lines in vitro

**Figure 4:**
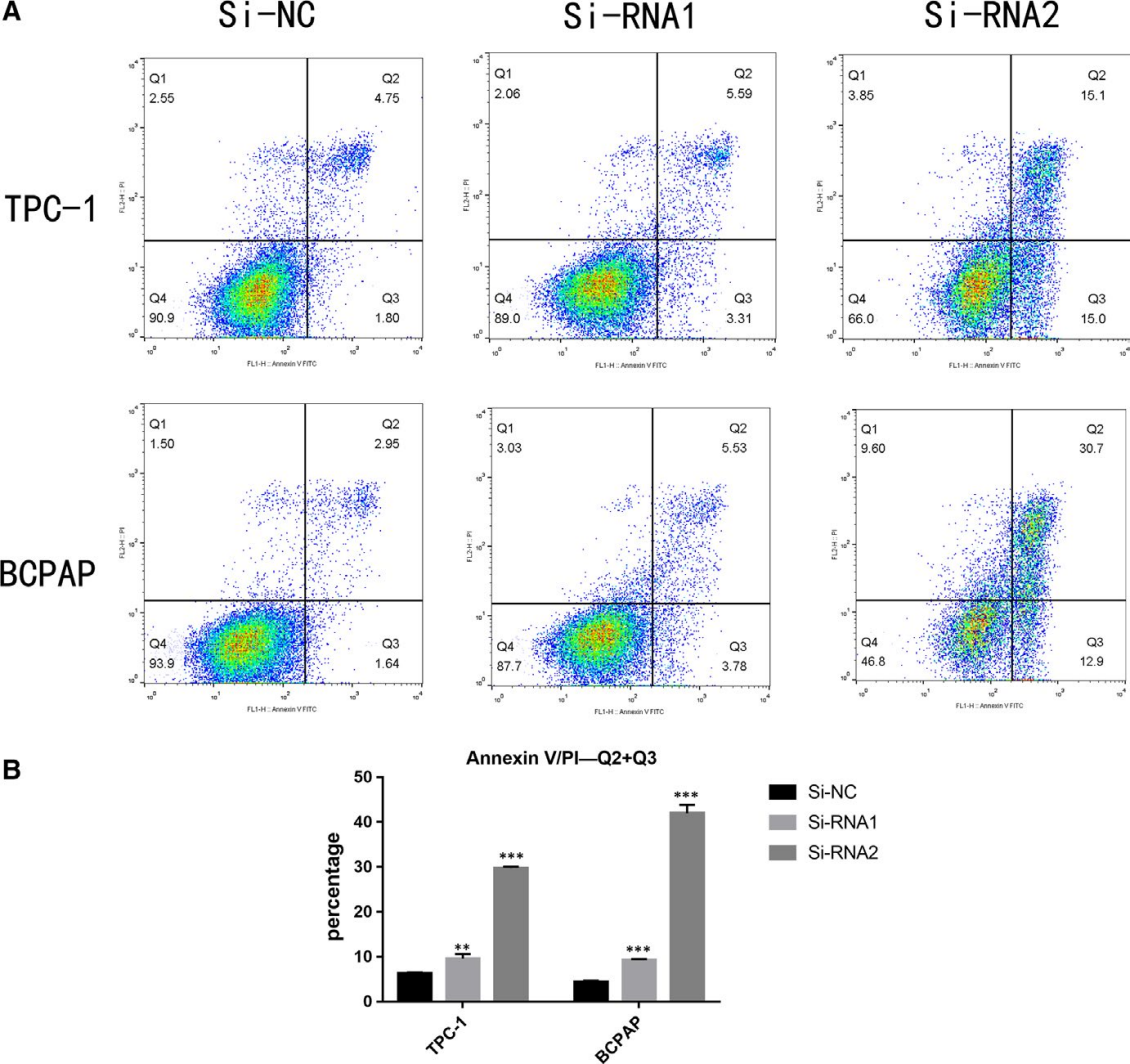
Growth‐associated protein 43 (GAP43) gene knockdown induces the cell apoptosis of papillary thyroid carcinoma cell lines in vitro. A, Annexin V/PI assay was applied to analyse the TPC‐1 and BCPAP cell lines transfected with Si‐NC or Si‐RNA1 or Si‐RNA2. Knocking down GAP43 could increase apoptotic cell death in thyroid cancer (TC) cells. B, Relative quantification of the apoptosis cell number. Q2 + Q3 was regarded as a set of standard to measure the cell apoptosis. Down‐regulation of GAP43 could increase much more quantification of Q2 + Q3 in TC cells. The columns represent the mean of death cell numbers from at least three independent experiments. **P < 0.01; ***P < 0.001 in comparison with si‐NC using Student's *t*‐test

We presumed that GAP43 also plays a role in the cycle of cell, so flow cytometry was used to investigate the proportion of apoptotic cells transfected with si‐GAP43 cell lines. The results showed that knockdown of GAP43 induced increased apoptosis in TC cells (TPC1, BCPAP), especially late‐stage apoptotic cells, compared with corresponding Si‐NC cell lines (Figure 4A, 4B).

### 3.7 GAP43 modulates epithelial‐mesenchymal transition in thyroid carcinoma cell lines

**Figure 5:**
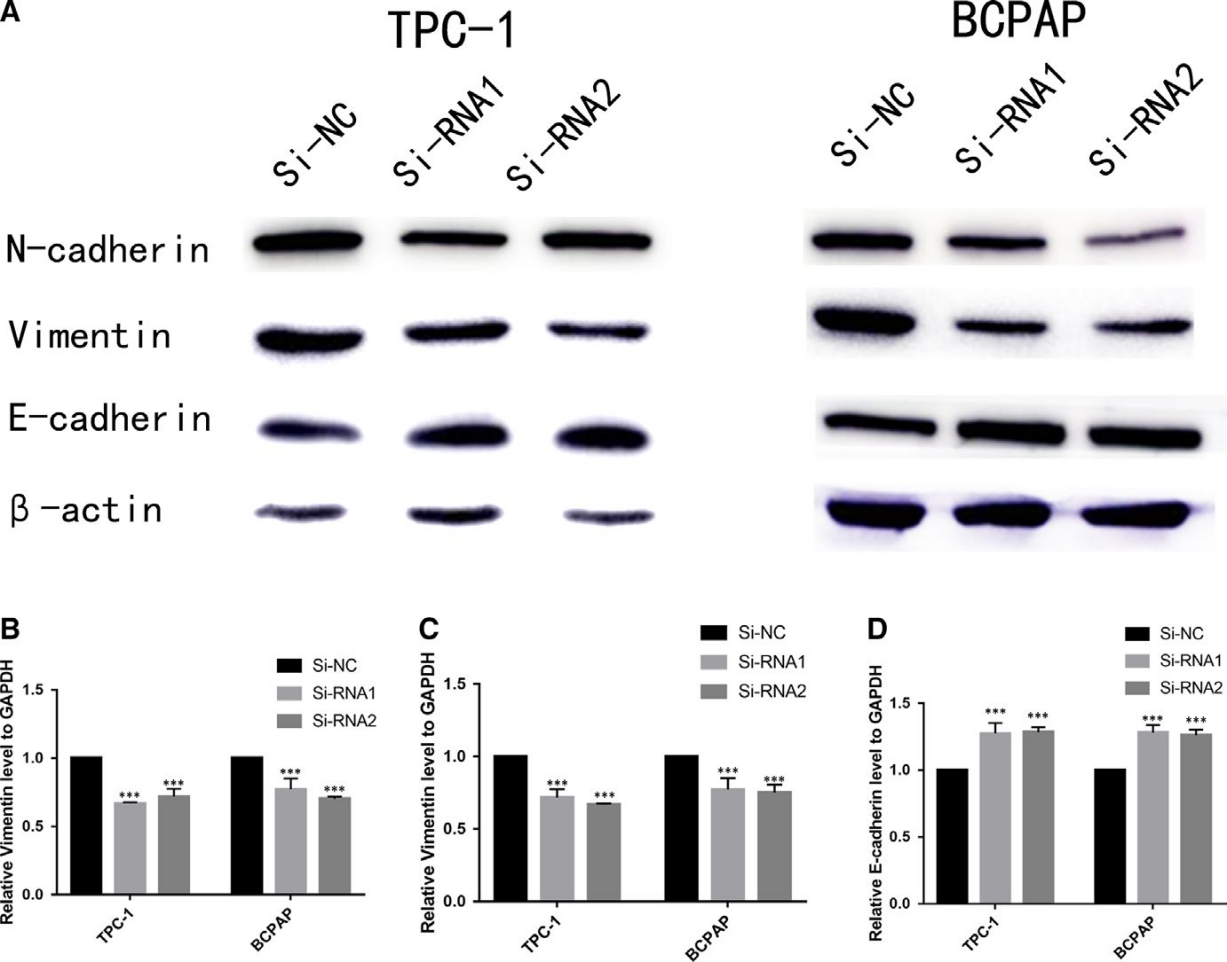
GAP43 modulates epithelial‐mesenchymal transition in thyroid carcinoma cell lines. A, Effect of GAP43 on epithelial‐mesenchymal transition proteins in thyroid carcinoma cell lines (TPC‐1 and BCPAP). E‐cadherin, N‐cadherin and vimentin protein expression determined by Western blotting in transfected TPC‐1 and BCPAP cells. B‐D, Relative quantification of densitometric analysis. Knockdown of GAP43 could increase protein level of E‐cadherin and decrease expression of N‐cadherin, and vimentin in transfected TPC‐1 and BCPAP cells. ***P < 0.001. Abbreviations: GAP43, growth‐associated protein 43; si‑NC, siRNA negative control; siRNA, small interfering RNA

Accumulating studies reported that epithelial‐mesenchymal transition (EMT) is a critical process especial during cancer cell metastasis.^26^, ^27^, ^28^ As shown in Figure 5A‐D, the expression of vimentin and N‐cadherin was deceased and expression of E‐cadherin was enhanced in si‐GAP43 cell lines. Those results suggested that GAP43 may promote thyroid cell metastasis by changing EMT.

## 4 DISCUSSION

Thyroid cancer, as one of the quickly growing malignant tumour, is expected to surpass colorectal cancer and became the fourth most commonly diagnosed cancer in 2030.^29^ Accumulating evidence has proved that GAP43 has a crucial function in the process of neural cell growth and development, but the underlying molecular mechanisms of GAP43 in thyroid carcinoma are still poorly understood.

Next‐generation sequencing has been widely used in the study of the gene expression variations and cancer molecular mechanism. In our previous study,^19^ we selected 19 matched pairs of thyroid carcinoma tissues and nearby non‐cancerous tissues and the outcomes revealed that GAP43 expression was overexpressed in tumour tissues. This finding suggested GAP43 may have a possible role as an oncogene in PTC.

In both TCGA and local cohorts of PTC, the clinicopathologic feature analysis showed that the overexpression of GAP43 was significantly related to the aggressive clinicopathologic characteristics, such as tumour size and LNM. Furthermore, knockdown of GAP43 could repress PTC cell proliferation and colony formation, as well as migration and invasion capacities of PTC cells. We also showed that underexpression of GAP43 may inhibit protein level of N‐cadherin and vimentin and enhance E‐cadherin. This discovery suggests that GAP43 can regulate the metastasis of PTC cell lines by modulating EMT. To the best of our knowledge, this study is firstly investigated the significant function of GAP43 in PTC.

Despite these exciting findings, some limitations still exist in this study. The relationship between GAP43 and the prognosis of PTC should be studied in large samples. In addition, animal experiments should be performed to test and verify the oncogenic function of GAP43.

In a summary, the present study investigated the association between GAP43 and clinicopathological features in two PTC cohorts (TCGA and local). Additionally, we showed that GAP43 knockdown impairs TC cell proliferation, colony formation, migration, invasion and induces cell apoptosis via EMT pathway. These interesting findings provide a potential molecular marker of PTC for diagnosis and therapy.

## CONFLICT OF INTEREST

Above authors declare that they have no conflict of interest.

## AUTHOR CONTRIBUTIONS

Chen Zheng did the main experiments and wrote the manuscript. Rui‐da Quan and Cheng‐Yong Wu help gathered and analyzed the raw data. Adheesh Bhandari helped to revise the article. Xiao‐hua Zhang conceptualized and designed the study and provided supervision.

## Data Availability

The data sets supporting the conclusions of this study are included in this article and its additional images. Raw data are available on the main electronic data storage system of First Affiliated Hospital of Wenzhou Medical University and access can be provided upon request to the authors.
